# Asiaticoside Mitigates Alzheimer’s Disease Pathology by Attenuating Inflammation and Enhancing Synaptic Function

**DOI:** 10.3390/ijms241511976

**Published:** 2023-07-26

**Authors:** Sai Liu, Long Chen, Jinran Li, Yuan Sun, Yue Xu, Zhaoxing Li, Zheying Zhu, Xinuo Li

**Affiliations:** 1 State Key Laboratory of Natural Medicines, China Pharmaceutical University, Nanjing 210009, China; 2 Jiangsu Provincial Key Laboratory of Drug Metabolism and Pharmacokinetics, China Pharmaceutical University, Nanjing 210009, China; 3 Division of Pharmaceutics and Pharmacology, College of Pharmacy, The Ohio State University, Columbus, OH 43210, USA; 4 State Key Laboratory of Natural Medicines and Jiangsu Key Laboratory of Drug Design and Optimization, China Pharmaceutical University, Nanjing 210009, China; 5 School of Pharmacy, The University of Nottingham, Nottingham NG7 2RD, UK

**Keywords:** Alzheimer’s disease, Asiaticoside, synapse, inflammation, p38 MAPK

## Abstract

Alzheimer’s disease (AD) is a prevalent neurodegenerative disorder, hallmarked by the accumulation of amyloid-β (Aβ) plaques and neurofibrillary tangles. Due to the uncertainty of the pathogenesis of AD, strategies aimed at suppressing neuroinflammation and fostering synaptic repair are eagerly sought. Asiaticoside (AS), a natural triterpenoid derivative derived from Centella asiatica, is known for its anti-inflammatory, antioxidant, and wound-healing properties; however, its neuroprotective function in AD remains unclear. Our current study reveals that AS, when administered (40 mg/kg) in vivo, can mitigate cognitive dysfunction and attenuate neuroinflammation by inhibiting the activation of microglia and proinflammatory factors in Aβ_1-42_-induced AD mice. Further mechanistic investigation suggests that AS may ameliorate cognitive impairment by inhibiting the activation of the p38 MAPK pathway and promoting synaptic repair. Our findings propose that AS could be a promising candidate for AD treatment, offering neuroinflammation inhibition and enhancement of synaptic function.

## 1. Introduction

Alzheimer’s disease (AD), a widely recognized neurodegenerative disorder mainly affecting the elderly, is a leading cause of dementia, imposes substantial medical costs, and consumes extensive caregiving resources. A threefold escalation in the global prevalence of dementia by 2050 has been predicted [1]. AD is typified by synapse degeneration, primarily within the neocortex, accompanied by the accumulation of senile plaques comprising predominantly amyloid-beta (Aβ) deposits, as well as a neuronal element characterized by the proliferation of hyperphosphorylated tau proteins, neurofibrillary tangles, and neuroinflammation [2,3].

Neuroinflammation, a key player in the onset and progression of AD, has been demonstrated to accentuate disease severity via the production of proinflammatory factors [4,5,6]. This response is typically instigated upon the activation of microglia by Aβ [7,8]. A multitude of epidemiological studies have provided evidence to suggest that anti-inflammatory therapies may serve to reduce the risk of AD [9,10]. For instance, the inhibition of p38 mitogen-activated protein kinases (p38 MAPK), which are involved in the activation of microglia and produce a wide range of proinflammatory factors such as IL-1β and IL-6, alleviates AD pathologies [11,12].

Emerging evidence increasingly suggests that mild synaptic impairment occurs at the initial stages in Alzheimer’s patients, predating the onset of neuronal degeneration [13]. Several studies have suggested that the synaptic dysfunction appears to be caused by diffusible oligomeric assemblies of the Aβ [14,15]. Further studies have established that the neuroinflammation can induce synaptic abnormalities by altering synaptic proteins [16,17]. These synaptic abnormalities could contribute to cognitive and memory impairment, which are prominent symptoms of AD [18,19]. Thus, inhibition of neuroinflammation might ameliorate the loss of synaptic proteins, subsequently improving cognitive and memory deficits in AD patients. Therefore, this approach may provide a promising and efficient treatment for AD [20].

The mitogen-activated protein kinases (MAPKs) comprise a family of serine and threonine protein kinases, which are expressed in both neuronal and non-neuronal cells within the mature central nervous system (CNS) [21]. These kinases are crucial in various cellular processes, encompassing cell proliferation, differentiation, and survival [22]. To date, more than a dozen MAPK enzymes have been identified, with the most well-characterized being the extracellular signal-regulated kinases 1 and 2 (ERK1/2), ERK5, c-Jun amino-terminal kinases 1 to 3 (JNK1 to 3), and the P38 (α, β, γ, and δ) families [23]. Regarding the pathophysiology of AD, numerous studies have indicated that the p38 MAPK cascade becomes activated in response to the Aβ peptide. This activation prompts the amplification of inflammatory responses through the synthesis and release of proinflammatory cytokines, culminating in synaptic deficit [24]. Moreover, a study has demonstrated that the inhibition of p38 MAPK can help alleviate memory impairment and neuroinflammation in AD mouse models [25].

The currently clinically used drugs to treat AD primarily act on cholinergic or glutamatergic neurotransmission, offering only symptomatic relief by slowing the disease’s progression rather than halting or reversing its pathological process [26]. Given the limitation of current treatments, there is a huge clinically unmet need to discover novel therapeutic drugs or strategies for the improved treatment of AD [27]. Therefore, new approaches focusing on anti-inflammation and promoting synaptic repair have garnered significant attention.

Asiaticoside (AS), a triterpenoid derivative extracted from Centella asiatica [28], manifests a broad spectrum of pharmacological activities, encompassing angiogenesis, anti-inflammation, osteogenic differentiation, and neuroprotection [9,29]. Previous studies have demonstrated that AS can inhibit the hyperactivation of hippocampal microglial cells and the phosphorylation of p38 MAPK, thereby exerting potent anti-neuroinflammation and neuroprotective effect [30,31]. Furthermore, AS may attenuate Aβ-induced cellular growth inhibition and counteract the neurotoxicity exerted by Aβ, suggesting a potential role for AS in anti-neuroinflammation and neuroprotection interventions in AD [30].

Inflammatory processes associated with synaptic plasticity impairment underpin the memory deficits that are a characteristic of AD [7]. Therefore, there is an essential need for further research to investigate whether AS can effectively mitigate the impacts of AD, particularly through mechanisms associated with synaptic regeneration and anti-inflammation. In this study, we seek to bridge this gap by investigating the therapeutic potential of AS for AD, focusing on its role in mitigating cognitive dysfunction. This study will provide an insight into the mechanism underlining AS therapeutics for AD, thereby providing evidence for potential clinical applications.

## 2. Results

### 2.1. AS Mitigated Learning and Memory Impairments in Aβ_1-42_-Induced Mice

**Figure 1:**
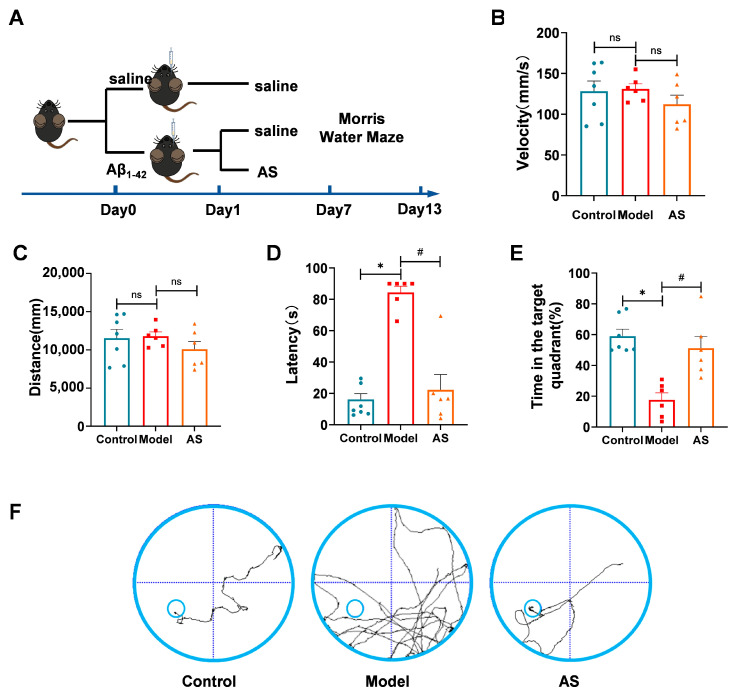
AS ameliorated learning and memory deficits in Aβ_1-42_-induced mice. Mice were injected with Aβ_1-42_ (2 μg/μL, 5 μL, i.c.v.) followed by AS treatment (40 mg/kg, i.g.). (**A**) Schematic representation of the experimental design. (**B**) Average swimming speed for each experimental group. (**C**) Distance traversed by different groups during the test. (**D**) Latency to locate the target platform during the final exploration trial. (**E**) Time spent in the target quadrant during the last exploration trial (**F**) Swimming paths of mice to target the hidden platform during the final exploration trial. Data are presented as mean ± SEM (*n* ≥ 3). * *p* < 0.05, model vs. control group; ^#^ *p* < 0.05, AS vs. model group.

To explore the potential therapeutic efficacy of AS on cognitive function, we conducted the Morris water maze test to evaluate the influence of AS on learning and memory abilities (Figure 1A) in Aβ_1-42_-induced mice. To exclude potential confounding effects from movement disorders on spatial learning, we recorded the distance and average swimming speed during the Morris water maze test (Figure 1B,C). Our findings revealed that all the mice exhibited normal motility. However, Aβ_1-42_-induced mice required more time to locate the platform compared to the control group, indicating a marked cognitive decline induced by Aβ_1-42_ (Figure 1D). Furthermore, a significant increase in escape latency was observed in AS-treated Aβ_1-42_-induced mice, signifying that AS treatment substantially enhanced cognitive function and learning ability.

To evaluate the influence of AS on spatial memory ability, probe trials were performed. Aβ_1-42_-induced mice treated by AS showed a significant increase in time spent in the target quadrant (Figure 1E), which demonstrates amelioration of spatial memory ability. Furthermore, trajectories of mice monitored during the final trial demonstrated that AS-treated Aβ_1-42_-induced mice needed less swimming time and shorter swimming distance to find the platform than Aβ_1-42_-induced mice, which suggested that AS may improve the spatial memory ability of Aβ_1-42_-induced mice (Figure 1F). Taken together, these results indicated that AS could ameliorate cognitive dysfunction in Aβ_1-42_-induced mice.

### 2.2. AS Exerted Anti-Inflammatory Effects in Aβ_1-42_-Induced Mice

**Figure 2:**
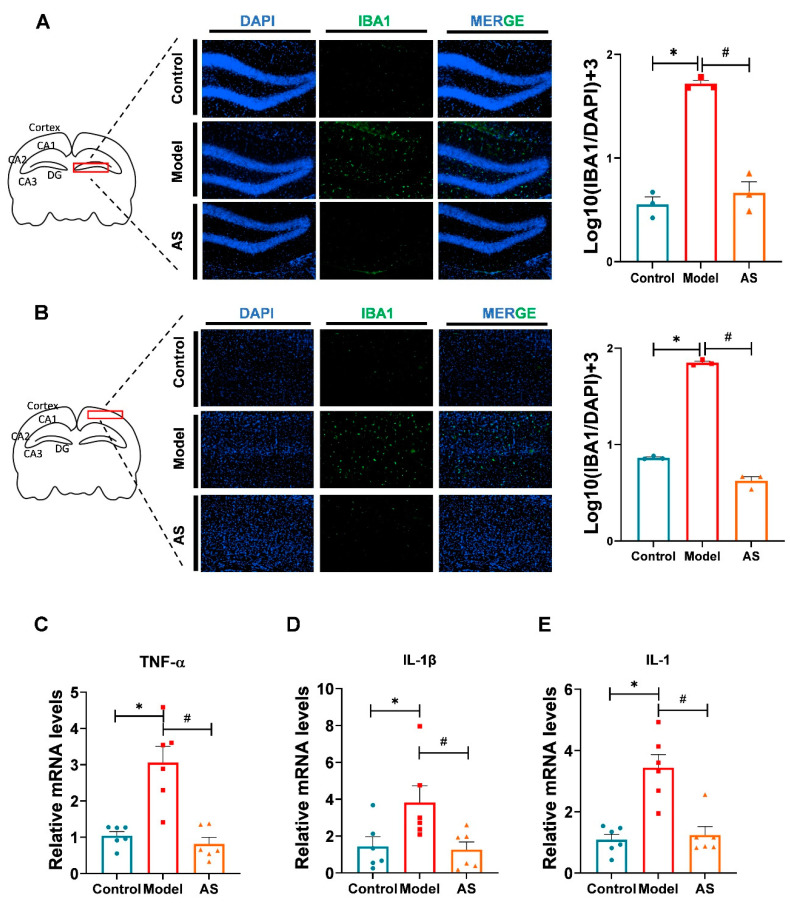
AS inhibited microglia activation and exerted antiinflammation in Aβ_1-42_-induced mice. (**A**) Representative fluorescence micrographs showing IBA1 expression in the hippocampus (scale bar, 200 μm), and quantification of the total number of IBA1^+^ cells in the hippocampus (*n* = 3). (**B**) Representative fluorescence micrographs showing IBA1 expression in the cortex (scale bar, 200 μm), and quantification of the total number of IBA1^+^ cells in the cortex. (*n* = 3). (**C**) The mRNA expression level of TNF-α was detected by real-time PCR. (**D**) The mRNA expression level of IL-β was detected by real-time PCR. (**E**) The mRNA expression level of IL-1 was detected by real-time PCR. Data are shown as mean ± SEM (*n* ≥ 3). * *p* < 0.05, model vs. control group; ^#^ *p* < 0.05, AS vs. model.

Studies have confirmed that Aβ_1-42_ plaques bind to microglia surface receptors, promoting microglia activation and subsequently releasing proinflammatory cytokines in the brain of mice [32]. This process largely contributes to the severity of AD [20]. To ascertain whether AS could exert anti-inflammatory effects in Aβ_1-42_-induced mice, we performed immunofluorescence in microglia of the mouse brain. The results revealed that AS could inhibit microglia activation caused by Aβ_1-42_ in both the cortex and hippocampus (Figure 2A,B). Additionally, we assessed the relative mRNA levels of proinflammatory factors (Figure 2C,E). The data showed that compared to the Aβ_1-42_-induced mice, the proinflammatory factors, including TNF-α, IL-β and IL-1 were relatively downregulated by AS treatment. Our results indicated that AS could exert anti-inflammatory effects in AD mice through the inhibition of microglial activation and the expression of proinflammatory factors at mRNA levels.

### 2.3. AS Promoted Synaptic Repairment and Improved Synapse Function in Aβ_1-42_-Induced Mice

**Figure 3:**
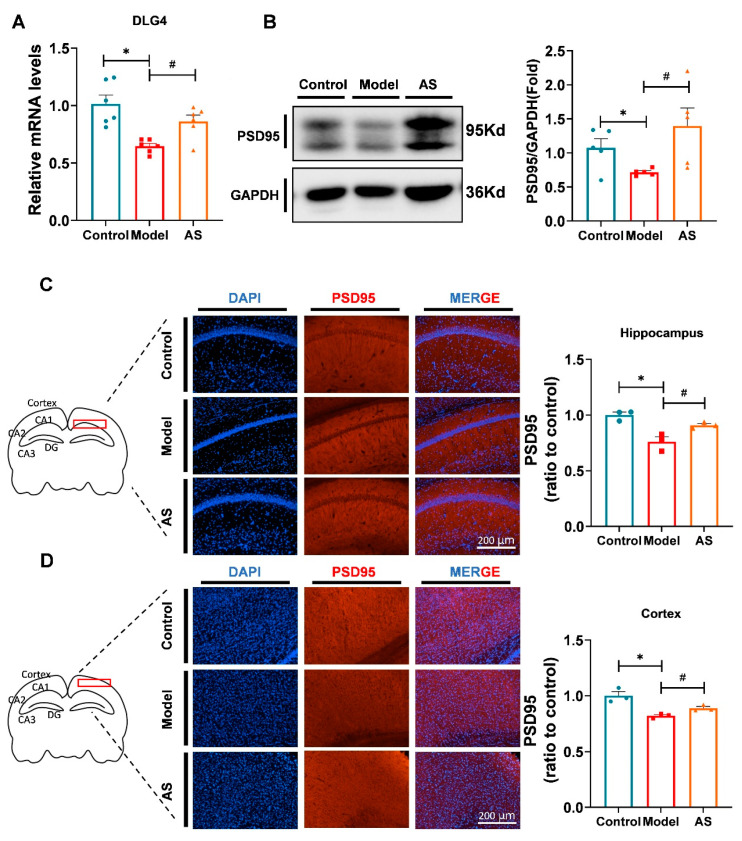
AS promoted synaptic repair in the brains of Aβ_1-42_-induced mice. (**A**) Quantified image showing the mRNA expression level of Dlg4. (**B**) Western blot analysis of PSD95 protein level in the brain. (**C**) Representative fluorescence micrographs showing PSD95 expression in the hippocampus (scale bar, 200 μm), and quantification of the total number of PSD95^+^ cells in the hippocampus. (*n* = 3). (**D**) Representative fluorescence micrographs showing PSD95 expression in the cortex (scale bar, 200 μm), and quantification of the total number of PSD95^+^ cells in the cortex. (*n* = 3). Data are shown as mean ± SEM (*n* = 3). * *p* < 0.05 vs. control group; ^#^ *p* < 0.05 vs. Aβ_1-42_-induced group.

The coactivation of proinflammatory cytokines and cytotoxic products during neuroinflammation damages neurons by altering synaptic proteins [7,20]. Several studies have indicated that synaptic deficiency is a critical cause of memory impairment in AD brains, and promoting synaptic repair is a potential strategy for mitigating or treating AD [2]. To verify whether AS could ameliorate synaptic dysfunction, we selected PSD95, encoded by Dlg4 [33], as an indicator of synaptic damage. The mRNA expression level of Dlg4 was found to be upregulated by AS treatment (Figure 3A), and the protein expression of PSD95 indeed increased in the brain of AD mice treated by AS (Figure 3B–D). Our data suggested that AS could improve synaptic dysfunction in Aβ_1-42_-induced mice. Given the anti-inflammatory effect of AS, it may exert synaptic protection by inhibiting microglial activation and inflammation. However, the potential molecular mechanism underlying this effect warrants further investigation.

### 2.4. AS Altered Genes Related to the p38 MAPK Pathway and Synaptic Function

**Figure 4:**
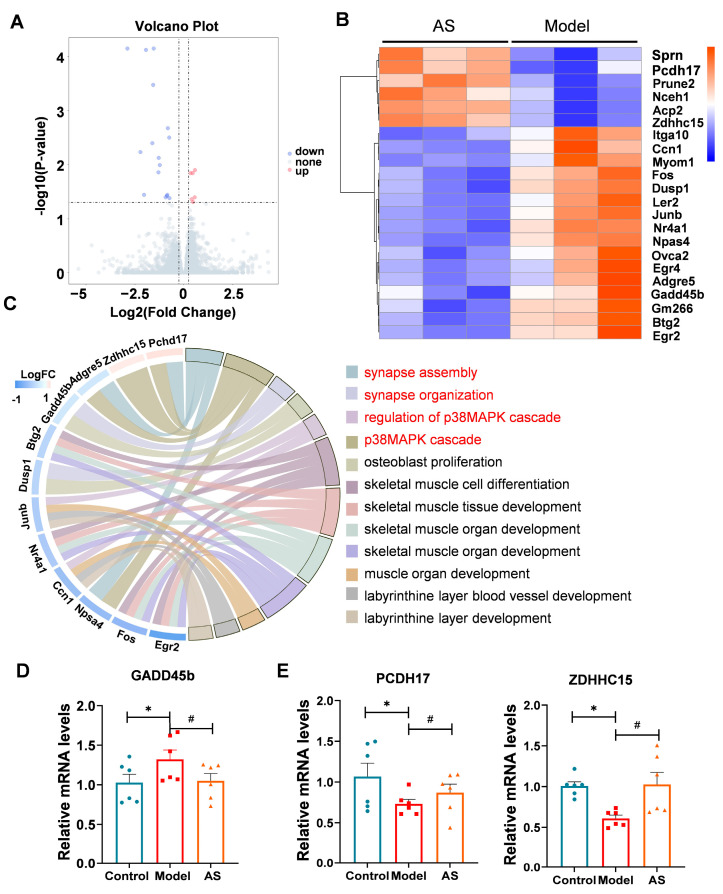
AS modulated mRNA expression profile in Aβ_1-42_-induced mice. (**A**) Volcano plot of differentially expressed genes (DGEs) in AS-treated Aβ_1-42_-induced mice compared to Aβ_1-42_-induced mice. (**B**) Heatmap of DGEs in AS-treated Aβ_1-42_-induced mice and Aβ_1-42_-induced mice. (**C**) Chord diagram illustrates the relationship between the investigated gene and its associated pathway. (**D**) The mRNA expression level of GADD45b was detected by real-time PCR. (**E**) The mRNA expression levels of Pcdh17 and Zdhhc15 were detected by real-time PCR. Data are presented as mean ± SEM (*n* ≥ 3). * *p* < 0.05, model vs. control; ^#^ *p* < 0.05, AS vs. model.

To elucidate the underlying mechanism of AS’s therapeutic impact, we analyzed brain samples using RNA sequencing. Setting the corrected adjusted *p*-value at <0.05 and log2 fold change at >0.58 as the threshold, we identified a total of 22 differentially expressed genes (DEGs) between the two groups, including 6 upregulated genes and 16 downregulated genes (Figure 4A,B). Subsequent gene ontology analysis revealed that the AS affected various pathways [34] (Figure 4C), mainly including the p38 MAPK pathway, mediated by Dusp1 and Gadd45b. Gadd45b, the growth arrest and DNA-damage-inducible gene, can promote adipose inflammation in mice [35], and its decrease may result in the inhibition of the p38 MAPK pathway. Our data showed that AS could downregulate Gadd45b in Aβ_1-42_-induced mice (Figure 4D), indicating that the anti-inflammation effect of AS may be attributed to the inhibition of the p38 MAPK pathway.

Synapse damage can occur due to the interaction between synaptic proteins and proinflammatory factors. Interestingly, AS modulated signaling pathways related to synaptic function regulated by Zdhhc15, Pcdh17, Npas4 and Adgre5 (Figure 4B). Indeed, RT-PCR results confirmed that AS could upregulate the expression of Pcdh17 and Zdhhc15 (Figure 4E). Pcdh17 (protocadherin-17) mediates collective axonal elongation by recruiting the actin-regulatory complex to the intraexon [36]. Additionally, Zdhhc15, a type of palmitoyl transferase, regulates dendrite morphology and excitatory synaptic formation. Zdhhc15 knockdown reduces palmitoylation of PSD95 and its transport to dendrites, resulting in an overall decrease in the density of excitatory synapses formed on mutant cells [37]. Thus, the upregulation of Pcdh17 and Zdhhc15 induced by AS treatment could contribute to synaptic repair. Collectively, our results suggest that AS may exert its anti-inflammatory effect by downregulating the Gadd45b-mediated p38 MAPK signaling pathway, leading to synaptic repairment.

**Figure 5:**
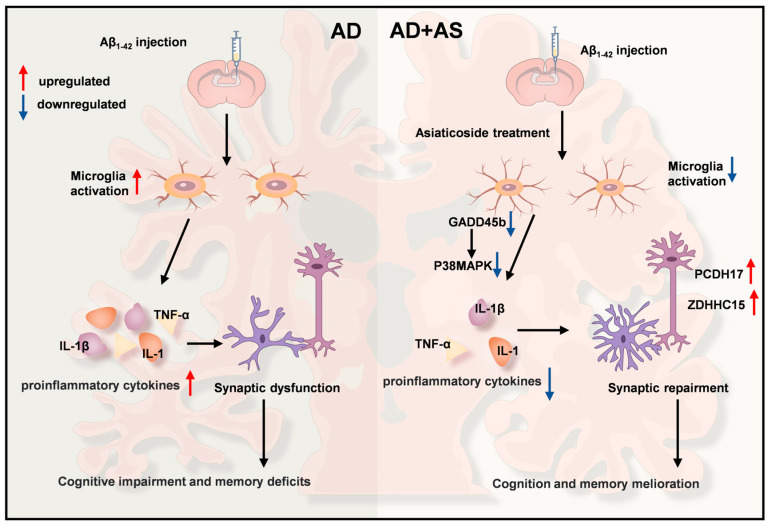
Schematic of the mechanism of AS improving synaptic impairment and cognitive impairment in Aβ_1-42_-induced mice. AS may improve cognitive dysfunctions by modulating the p38 MAPK pathway, resulting in the inhibition of proinflammatory factors production and improvement of synapse function, which in turn promotes improvement of cognitive function in AD mice.

In summary, our results indicated that AS might ameliorate cognitive and memory deficits by downregulating the p38 MAPK pathway and inhibiting neuroinflammation in Aβ_1-42_-induced mice. Conversely, the enhancement of synaptic repair, simultaneously with the inhibition of neuroinflammation, appears to improve cognition and memory melioration in AD mice (Figure 5).

## 3. Discussion

Our findings indicated that AS could enhance the learning and memory ability of Aβ_1-42_-induced mice, with further investigation suggesting that AS significantly inhibits microglia activation and proinflammatory factors in the hippocampus and cortex. Our data confirmed that AS could promote synaptic repairment in Aβ_1-42_-induced mice. Furthermore, RNA sequencing revealed that AS might modulate the p38 MAPK pathway and stimulate synaptic repairment, thereby confirming the protective effect of AS in AD. Overall, this study implied that AS has a promising perspective as a therapeutic agent for treating or mitigating AD.

AD is histopathologically characterized by Aβ plaque deposition, hyperphosphorylated tau protein, neuroinflammation, and synaptic loss, with clinical symptoms manifesting as progressive memory loss and cognitive dysfunction [38]. Neuroinflammation, a central factor, plays a significant role in the onset and progression of AD [19]. Numerous studies have reported that anti-inflammatory therapeutic strategies in the brain can ameliorate memory impairment and synaptic damage in AD [9,39]. Additionally, a wealth of research has demonstrated that AS can inhibit neuroinflammation and protect neurons by alleviating the neurotoxicity exerted by Aβ [29,30,31]. Furthermore, our result showed that AS could inhibit microglia activation and the production of proinflammatory factors, indicating that AS may serve as a potential therapeutic agent for AD through its neuroinflammatory alleviation.

Prior studies have demonstrated that the activation of p38 MAPK occurs in the postmortem brains of AD patients and animal models, leading to neuroinflammation [11,40]. Inhibition of p38 MAPK effectively alleviates neuroinflammation and synapse impairment in AD [24,41]. RNA sequencing in our study revealed that AS alters the p38 MAPK pathway and promotes synaptic repair in Aβ_1-42_-induced mice. Gadd45b and Dusp1, involved in the activation of p38 MAPK [35,42,43], were altered by AS treatment, indicating that the anti-neuroinflammatory effect of AS may be attributed to the inhibition of the p38 MAPK pathway, a hypothesis that warrants further investigation.

Several studies have demonstrated that neuroinflammation can result in synaptic damage, which appears to occur at the early stage in AD and intensifies as the disease progresses [13,39]. Our data showed that AS could ameliorate synapse dysfunction, as confirmed by the upregulation of PSD95 induced by AS treatment. The effect of the synaptic repair may be attributed to the inhibition of neuroinflammation. RNA sequencing suggested that several genes related to synaptic repair were modulated by AS, indicating that AS may regulate the progress of promoting synaptic repair in Aβ_1-42_-induced mice. In our study, AS modulated Pcdh17, Npas4, and Zdhhc1, which regulate the growth and development of synapses [36,44], resulting in synaptic repair in Aβ_1-42_-induced mice. Overall, RNA sequencing indicated that AS might promote synaptic repair and inhibit neuroinflammation by regulating the expression of several genes related to synapse function and the p38 MAPK pathway.

Currently, FDA-approved drugs that target cholinergic or glutamatergic neurotransmission are available treatments for AD. However, these drugs only relieve symptoms, and do not reverse the progress of AD [26]. Previous studies have demonstrated that reducing inflammation can be a supplemental strategy for treating AD [27] in addition to the two treatments approved by FDA. AS, which can exert anti-inflammatory effects in the brain and improve synaptic loss and cognitive dysfunction, is a potential candidate compound as a single drug or a combination drug to treat AD.

Nevertheless, some limitations of this study warrant acknowledgment. The delivery of AS across the blood-brain barrier presents a significant challenge due to its large molecular weight and high topological polar surface area [45]. This impediment could be circumvented by formulating AS into a suitable delivery system such as liposomes or lipid nanoparticles [46]. Moreover, the precise molecular mechanism underlying the ability of AS to alleviate neuroinflammation and promote synaptic repair necessitates further elucidation.

In summary, our study revealed that AS could ameliorate cognitive dysfunction in Aβ_1-42_-induced mice, an effect attributed to its capacity to inhibit neuroinflammation and enhance synaptic repair. Our findings enrich the understanding of the beneficial impacts of AS in AD treatment, indicating that AS may hold promise as a potential therapeutic agent for AD, either as a standalone treatment or in combination with currently approved drugs.

## 4. Materials and Methods

### 4.1. Animals and Manipulations

Male C57/BL6 mice (6 weeks old, weight 18–22 g) were obtained from Changzhou Cavens Laboratory Animal Company (Changzhou, China). The animal culture and experiments were performed in line with the ethics guidelines of the Ministry of Science and Technology of the People’s Republic of China and approved by the Pharmaceutical Laboratory Animal Center of China Pharmaceutical University.

### 4.2. Aβ_1-42_ Injection and Drug Intervention

Recombinant human Aβ_1-42_ peptide was purchased from Beyotime Biotechnology (Shanghai, China). The Aβ_1-42_ peptide was dissolved in sterile PBS at 2 mg/mL and incubated at 37 °C for 5 days to induce Aβ_1-42_ aggregation [47,48]. All mice were divided into three groups (*n* = 8 in each group) comprising a blank control group, an Aβ_1-42_ model group, and an Aβ_1-42_ + AS (i.g., 40 mg/kg) group. On the first day of the experiment, mice were injected with 5 μL Aβ_1-42_ protofibrils into the lateral ventricle via brain stereo locator. The control group was injected with 5 μL saline in the same way. AS (dissolved in 5% CMC-Na) was administered intragastrically within 2–14 days. Mice in the control group and the model group were only given normal saline intragastric administration.

### 4.3. Morris Water Maze (MWM)

The MWM is commonly used to assess spatial learning and memory in mice. The Morris water maze test was conducted in a circular swimming pool (60 cm radius, 45 cm height) filled with water at a temperature of 25.0 ± 1 °C. The swimming pool was randomly divided into four quadrants, with a platform located in the third quadrant. The water was made opaque by adding titanium dioxide in order to facilitate tracking the movement trajectory of the mice. In addition, the pool was surrounded by many cues, and the location of the cues remained constant throughout the water maze task.

Behavioral tests were performed on the 7th day after treatment, and spatial learning and memory ability tests were performed on the 6th day after 5 days of training. Mice were placed on the opposite side of the quadrant from the platform facing the pool wall, and allowed to swim to the platform, which was placed 1 cm above the water surface. If the mouse reached the platform within 90 s, it was allowed to stay for 10 s. Otherwise, it was manually guided to the platform and kept on the platform for 10 s to allow it to remember the position. On the 6th day of the experiment, the platform was hidden 1 cm below the surface and the mice were tested for space exploration. Latency, path length, swimming speed, target quadrant residence time, and travel path were recorded daily by the computer.

### 4.4. Brain Tissue Preparation and Mice Brain Slices

The mice were killed under deep anesthesia and cardiac perfusion was performed. The mice used for the western blot experiment or RNA extraction were perfused with PBS for 2 min. After perfusion, the mouse brains were removed and stored in a −80 °C refrigerator.

In addition, mice used for immunofluorescence received cardiac perfusion with PBS for 1 min followed by 4% paraformaldehyde (PFA) for 1 min. After perfusion, the mouse brains were soaked in PFA for fixation and stored at 4 °C. Brain tissue that had been fixed for 48 h was dehydrated by soaking it in a 30% sucrose solution for 48 h. The brain was cut into coronal sections 20 μm thick using a cryotome (Leica, CM1950) after sinking to the bottom, and the brain sections with intact hippocampus were stored in a cryogenic solution (PBS: ethylene glycol: glycerin = 5:3:2) and stored at −20 °C.

### 4.5. Immunofluorescence

The brain slices were washed 3 times with PBS for 5 min each time and then treated with 0.1% Triton X-100 (Beyotime Biotechnology, ST795, Shanghai, China) diluted with PBS for 20 min. Then, slices were placed in 5% BSA (Beyotime Biotechnology, ST023, Shanghai, China) diluted with PBS and sealed at room temperature for 1 h. After incubating overnight with the primary antibody [rabbit antibody PSD95 (Abcam, ab238135, Cambridge, UK, 1:200), rabbit antibody IBA1 (Fujifilm, 019-19741, Tokyo, Japan, 1:300)] at 4 °C, the sections were washed 3 times with PBS. A secondary antibody [goat anti-rabbit IgG H&L (Alexa Fluor^®^ 488) (Abcam, ab150077, 1:500)] was then applied at room temperature for 1 h, and the slices were washed in PBS 3 times. Finally, the nuclei were restained with 1 μg/mL DAPI (Beyotime Biotechnology, C1002, Shanghai, China) for 10 min, and the slides were mounted with an anti-fluorescence quenching seal. The slides were viewed with a fluorescence microscope (BioTek, Cytation5, Hong Kong, China) and all images were collected using a microscopic imaging system (BioTek, Cytation5, Hong Kong, China). The ImageJ (v1.54f) application was used to analyze the collected images.

### 4.6. RNA Isolation and RNA Sequencing

The isolation of RNA from brains was conducted using the RNA isolation agent Total RNA Extraction Reagent (Vazyme, R401-01, Nanjing, China). First, 500 μL of the RNA isolation agent was mixed with 30 mg of brain sample and ground at 4 °C using a freeze grinder. After grinding, the samples were centrifuged at 11,200 rpm for 5 min at 4 °C. After being allowed to stand for 5 min, an emulsion, formed from the supernatant with the addition of 20% volume of chloroform by vigorous shaking for 15 s, was centrifuged at 11,200 rpm for 15 min at 4 °C. After the addition of an equal volume of isopropanol precooled at 4 °C, a colorless aqueous phase layer formed by centrifugation was reversed, mixed, and left for 10 min. The mixed solution was centrifuged at 11,200 rpm for 10 min at 4 °C, and the white precipitate was precipitated. The supernatant was discarded and 1 mL of 75% ethanol was added to suspend the precipitate, which was reversed up and down several times. The precipitate was left for 5 min and centrifuged at 11,200 rpm for 5 min at 4 °C, the supernatant was discarded, and the precipitate was dried in a clean environment at room temperature for 3 min. RNase-free ddH_2_O was added to dissolve the precipitate. The RNA extract was stored at −80 °C after determination of the concentration.

### 4.7. RNA-Seq Analysis

The isolated RNA was subsequently used for RNA-seq analysis. cDNA library construction and sequencing were performed by Frasergen Genomic Medicine (Wuhan, China). High-quality reads were aligned to the mouse reference genome using Hisat2 (v2.0.3). We identified DEGs between samples and performed clustering analysis and functional annotation using R studio (v 4.2.2).

### 4.8. Real-Time PCR

Total RNA isolated from brains was transferred to cDNA using HiScript RT SuperMix for qPCR (+gDNA wiper) purchased from Vazyme (Nanjing, China). For gene expression analysis, PCR amplification was performed using Taq Pro Universal SYBR qPCR Master Mix purchased from Vazyme and the samples were run on a CFX96™ Real-Time PCR Detection system (Bio-Rad, v2.2). The primers purchased from Invitrogen were designed using PrimerBank and Primer-BLAST (Supplementary Table S1). To calculate gene expression, the 2^−ΔΔCt^ method was used with GAPDH expression as a normalizer and an untreated sample as relative control.

### 4.9. Western Blotting

A RIPA lysis buffer was configured by mixing PMSF and RIPA (1:100). The mouse brains stored in a −80 °C refrigerator were taken out. A portion of the cortex of the brain was removed, weighed and added to the RIPA lysis buffer, and then the cortex tissue was ground using a freeze grinder. After grinding, the mixture was left on ice for 30 min and occasionally swirled. The mixture was centrifuged, the supernatant was absorbed, and the buffer was added at a ratio of 4:1. The mixture was then boiled at 100 °C using a constant-temperature mixing instrument for 15 min. After the above steps, we obtained a protein sample for western blotting. The protein sample was placed at room temperature and stored at −20 °C after cooling. Protein samples were separated by 10% sodium dodecyl sulfate-polyacrylamide gel electrophoresis (Bio-Rad, Hercules, CA, USA) and were then transferred to PVDF membranes (Bio-Rad, Hercules, CA, USA). After blocking with 5% nonfat milk in TBST (0.1% tween 20 in TBS), the membranes were probed with different primary antibodies overnight at 4 °C. The primary antibodies used were as follows: rabbit anti-PSD95 antibody (Abcam, 1:1000) and rabbit anti-synaptophysin antibody (Abcam, 1:5000). The membranes were washed 3 times with TBST, followed by washing with respective HRP-conjugated secondary antibodies for 1 h. After three more washes with TBST, protein bands were visualized using enhanced chemiluminescence (ECL) reagents (Tanon, Shanghai, China) and signals were captured using a Tanon-5200 Chemiluminescent Imaging System (Tanon Science & Technology, Shanghai, China). The intensities of the protein bands were quantified with ImageJ (v1.45f).

### 4.10. Statistical Analysis

At least three biological replicates were performed for each experiment to ensure consistency. Data were expressed as mean ± SEM, and Student’s *t*-test was used for statistical analyses. Statistical significance was set at a *p* value of less than 0.05.

## Data Availability

RNA-seq data that supports the findings of this study has been successfully deposited in NCBI with the accession number PRJNA976044 (SAMN35358035-SAMN35358040).

## Supplementary Materials

The following supporting information can be downloaded at: https://www.mdpi.com/article/10.3390/ijms241511976/s1.
