# Supplementary material for: Asiaticoside Mitigates Alzheimer’s Disease Pathology by Attenuating Inflammation and Enhancing Synaptic Function
Source: Int J Mol Sci. 2023 Jul 26;24(15):11976. doi: 10.3390/ijms241511976 (PMC10418370; doi:10.3390/ijms241511976)
Supplement: Supplementary file 1 [file ijms-24-11976-s001.zip › ijms-2468673-supplementary.pdf]

Gene	F/R	Sequence
Gapdh	F	TGGCCTTCCGTGTTCCTAC
	R	GAGTTGCTGTTGAAGTCGCA
Dlg4	F	TGAGATCAGTCATAGCAGCTACT
	R	CTTCCTCCCCTAGCAGGTCC
Gadd45b	F	TTGACATCGTCCGGGTATCAG
	R	GTCTCGGGCTTCGGTTGTG
Dusp1	F	TGTTGTTGGATTGTCGCTCCT
	R	TTGGGCACGATATGCTCCAG
Zdhhc15	F	GTGCCAGTGCTCGTTATTGTC
	R	CTTTTTCCGCTGGACTCAAAAC
Pcdh17	F	GATTTGAACGCCACTGATGCT
	R	GGTTGCCCTTAACGCGGAT

Supplementary Table S1. RT-PCR primers
